# Molecular and Cellular Analysis of Lipogems-Processed Lipoaspirates for Evaluating the Efficacy of Treatments in Regenerative Medicine

**DOI:** 10.1007/s12015-026-11094-9

**Published:** 2026-04-11

**Authors:** Sura H. A. Al Sammarraie, Nicola Alessio, Domenico Aprile, Beatrice Castiglioni, Tiziana Squillaro, Michela Bosetti, Giovanni Di Bernardo

**Affiliations:** 1 Biotechnology and Molecular Biology Section, Department of Experimental Medicine, School of Medicine, University of Campania Luigi Vanvitelli, Naples, 80138 Italy; 2 Department of Life Sciences, Health and Health Professions, Link Campus University, Rome, 00165 Italy; 3 Dipartimento di Scienze del Farmaco, Università degli Studi del Piemonte Orientale, Novara, 28100 Italy; 4 Center for Biotechnology, Sbarro Institute for Cancer Research and Molecular Medicine, Temple University, Philadelphia, PA 19122 USA

**Keywords:** Lipoaspirate, Senescence, MSCs, Adipose tissue, Regenerative medicine, Pericytes

## Abstract

Autologous adipose tissue is a readily available source of mesenchymal stromal cells (MSCs) for regenerative applications. Here, human lipoaspirates from eight donors processed with the Lipogems system were characterized by flow cytometry (MSC/pericyte markers and inflammatory markers), immunocytochemistry (NANOG, OCT3/4, SOX2), and functional assays assessing proliferation, senescence, apoptosis, multilineage differentiation, IDO activity, and ROS levels. Lipogems-derived cultures fulfilled MSC immunophenotypic criteria while showing marked inter-donor variability in MSC/pericyte proportions, stemness-marker expression, differentiation bias, inflammatory profile, and oxidative status. In contrast, senescence and apoptosis were comparable across samples. Clinical evaluation, based on surgeon-assessed outcomes after autologous treatment, scored on a 1–10 scale, was consistently high across donors (7–10). Overall, Lipogems-processed lipoaspirates harbor a heterogeneous but functionally competent stromal compartment; these data support a sample-specific quality framework (composition, differentiation tendency, inflammation/ROS, IDO responsiveness) to improve reproducibility and optimization of regenerative protocols.

**Supplementary Information:**

The online version contains supplementary material available at 10.1007/s12015-026-11094-9.

## Introduction

Recent progress in stem cell research has opened new perspectives for regenerative and immunomodulatory therapies in clinical contexts where effective treatments remain limited. These include chronic inflammatory and immune-mediated disorders (such as inflammatory arthropathies and autoimmune conditions), degenerative musculoskeletal diseases (including osteoarthritis and tendon degeneration), and conditions characterized by impaired tissue repair, such as chronic wounds or post-traumatic defects [1–4]. In these settings, the therapeutic rationale increasingly relies not only on tissue replacement, but also on the modulation of inflammatory and immune responses, which play a central role in disease progression and regeneration.

Within this framework, mesenchymal stromal cells (MSCs) have attracted substantial interest due to their combined pro-regenerative and immunomodulatory properties [5]. Rather than acting primarily through direct differentiation in vivo, accumulating evidence indicates that MSCs exert their therapeutic effects mainly through paracrine signaling, secretion of bioactive factors, and regulation of innate and adaptive immune responses [6]. In line with this concept, MSC-based approaches have been investigated in a broad range of clinical indications, including autoimmune and inflammatory diseases, neurodegenerative disorders, and musculoskeletal injuries, with encouraging but variable outcomes.

Among the different MSC sources, adipose-derived stromal/stem cells (ADSCs) are particularly attractive because adipose tissue is abundant, easily accessible, and can be harvested in large quantities through minimally invasive autologous procedures. Lipoaspirates consist of fragments of subcutaneous adipose tissue that preserve a complex stromal architecture, including ADSCs, preadipocytes, pericytes, endothelial cells, and cells of hematopoietic origin [7–9]. The precise localization of stem and progenitor populations within adipose tissue remains a matter of investigation [7]; however, multiple histological and immunophenotypic studies support the existence of a perivascular niche, in which ADSCs closely interact with pericytes and endothelial cells, suggesting a continuum of vascular-associated mesenchymal progenitors [7, 10, 11].

According to established criteria, MSCs, including ADSCs, are typically defined by the expression of CD105, CD73, and CD90, and by the absence of hematopoietic and immune-related markers such as CD45, CD34, CD14/CD11b, CD79a/CD19, and HLA-DR [12]. Despite these consensus criteria, the stromal fraction obtained from adipose tissue is inherently heterogeneous, and only a subset of cells displays robust stemness and multilineage differentiation potential. This intrinsic variability represents a major challenge for the reproducibility and predictability of adipose-based regenerative therapies.

In regenerative and aesthetic medicine, several closed-system devices have been developed to harvest and process adipose tissue for autologous use [13]. The success of aesthetic and regenerative procedures depends critically on the choice of MSC source. The origin, biological properties, and cell yield of MSCs influence the secretion of growth factors, cytokines, extracellular vesicles, and other bioactive molecules that shape the regenerative microenvironment [9]. Selecting the most appropriate MSC source is therefore essential to harmonize these variables and achieve personalized, effective therapeutic outcomes. As a result, the clinical success of liposuction-based interventions, whether aesthetic or regenerative, largely depends on the biological quality and regenerative potential of the harvested adipose tissue [14]. In this context, characterizing the composition of the lipoaspirate before clinical use becomes crucial for predicting treatment success and minimizing variability in clinical outcomes. Several studies have documented substantial variability in cell yields obtained from different liposuction devices, likely due to the lack of standardized preparation protocols and analytical methods [14–16]. Even within individual studies, variability related to the harvesting site has been observed, emphasizing the influence of donor area [17]. Additional factors such as patient demographics, harvesting technique, and processed volume further contribute to inconsistent outcomes, limiting the reproducibility and comparability of published data [18, 19].

To mitigate these inconsistencies, several closed-system devices have been developed. Among them, Lipomed^®^, MiniTC^®^, Seffiller^®^, GenAdiposeTM, and Lipogems^®^ are the most commonly used platforms. The Lipogems system is widely employed and is based on a closed, enzyme-free mechanical process that gently micro-fragments adipose tissue while removing oil residues and blood components. This approach preserves the native stromal and perivascular architecture of the tissue, yielding microfragmented adipose clusters enriched in vascular-associated stromal elements, without extensive manipulation or in vitro expansion. Unlike methods aimed at isolating single-cell stromal vascular fractions, Lipogems is designed to maintain tissue integrity and cellular interactions that may be relevant for in vivo regenerative and immunomodulatory activity [13].

Despite the growing clinical use of Lipogems-processed lipoaspirates across multiple medical specialties, a detailed molecular and functional characterization of their cellular composition and biological properties remains limited. Several studies have reported substantial inter-donor variability in cell yield and functional performance, influenced by donor characteristics, harvesting site, and processing conditions. Such variability may contribute to inconsistent clinical outcomes and highlights the need for systematic analytical frameworks to better define tissue quality prior to therapeutic application.

In this study, we performed a comprehensive molecular and cellular characterization of human Lipogems-processed lipoaspirates, combining immunophenotypic, transcriptional, and functional analyses [20]. We evaluated proliferative capacity, senescence, apoptosis, stemness, multilineage differentiation potential, inflammatory and immunomodulatory markers, including indoleamine-2,3-dioxygenase (IDO), as well as intracellular reactive oxygen species (ROS) levels [21, 22]. By integrating these readouts, we aimed to capture intrinsic sample heterogeneity and to establish a multidimensional framework for predicting the regenerative and immunomodulatory potential of individual lipoaspirate preparations.

## Materials and Methods

### Patient Recruitment and Sample Collection

Human adipose tissue samples were obtained from adult donors undergoing elective orthopedic, regenerative, or aesthetic procedures involving autologous lipoaspiration. Lipoaspirate samples were obtained at the Image Regenerative Clinic (Milan, Italy). All procedures were performed under sterile conditions according to routine clinical practice.

Written informed consent was obtained from each participant prior to sample collection, specifying that residual biological material otherwise destined for disposal could be used for research purposes, in agreement with Recommendation Rec (2006) 4 of the Committee of Ministers of the Council of Europe on research on biological materials of human origin. The study protocol was reviewed and approved by the Institutional Ethical Committee of the University of Milan (C.E. UNIMI, approval number 59/25, #978).

Lipoaspirate was harvested from a single anatomical site (abdominal subcutaneous adipose tissue) from eight healthy donors (3 females and 5 males) undergoing elective liposuction, with a mean age of 52 ± 17.4 years (range 23–74 years) and a mean body mass index (BMI) of 23 ± 2.94 (range 17.3–32.7). Exclusion criteria included BMI > 35, diabetes, hypertension, and nicotine or alcohol abuse. Preoperative antibiotic prophylaxis was not administered, as all procedures were performed under sterile conditions.

After infiltration with 400 mL of saline solution containing adrenaline, adipose tissue was aspirated using a 10-cc syringe with a Luer-Lok^®^ tip connected to a disposable 19-cm blunt cannula (3 mm outer diameter) with five oval side holes (1 × 2 mm). Approximately 210 mL of lipoaspirate per patient were collected and processed using a Lipogems^®^ commercial device (Lipogems International S.p.A., Milan, Italy) according to the manufacturer’s instructions. Upon receipt, micronized adipose tissue was centrifuged (400 g, 5 min) to remove residual saline solution; a portion was cryopreserved at − 80 °C in 90% FBS and 10% DMSO as a backup for future analyses, while the remaining material was processed within 8 h for adipose-derived stromal cell (ADSC) isolation. The processed samples were then transported under controlled conditions to the Molecular Biology Laboratories of the University of Campania “Luigi Vanvitelli” (Naples, Italy), where all subsequent molecular and cellular analyses were performed as described below.

### MSC Isolation and Culture

Mesenchymal stromal cells (MSCs) were isolated from 300 mg of Lipogems-processed lipoaspirate. Samples were washed with phosphate-buffered saline (PBS) containing Ca²⁺ and Mg²⁺, then enzymatically digested in D-MEM supplemented with type II collagenase (1 mg/mL; Sigma, St. Louis, MI, USA) for 60 min at 37 °C. The digested suspension was filtered through a 70 μm cell strainer, centrifuged, and rinsed three times with PBS 1X (Microgem, Italy). The resulting cell pellet was plated in 100 mm dishes using low-glucose D-MEM (Microgem, Italy) supplemented with 10% Embryonic Stem cell–qualified Fetal Bovine Serum (ES-FBS, Euroclone, Italy), 5 ng/mL recombinant human basic fibroblast growth factor (PeproTech, UK), 1% penicillin/streptomycin, and 1% L-glutamine (Sigma-Aldrich, St. Louis, USA). In parallel, a commercially available adipose-derived MSC line (Sigma-Aldrich, St. Louis, USA) was cultured under identical conditions and used as a reference control throughout the study.

Cells were incubated for 7–10 days in proliferation medium until reaching confluence (passage 0, P0), then detached with trypsin (Sigma-Aldrich, St. Louis, USA) and expanded up to the third passage (P3).

### Flow Cytometry

MSCs were harvested by trypsinization and resuspended as single-cell suspensions, washed with PBS and incubated with fluorophore-conjugated antibodies: anti-CD105 Alexa Fluor 488 (Elabscience, Houston, TX, USA), anti-CD90 PE (Elabscience Houston, TX, USA), anti-CD73 APC (BioLegend, SAN, CA), anti-CD36 PE/Cy7 (BioLegend, San Diego CA), and anti-CD68 PE (BioLegend, SAN, CA). Antibody staining was performed according to the manufacturers’ instructions. After 30 min of incubation at room temperature in the dark, cells were washed with PBS and resuspended in FACS buffer. Data were acquired using a BD Accuri C6 flow cytometer (Becton Dickinson, NJ, USA) and analyzed with Accuri C6 software. Cell debris and doublets were excluded by FSC/SSC gating. At least 10,000 events per sample were collected and analyzed based on FSC versus SSC parameters.

### Immunocytochemistry

Cells were seeded on glass coverslips in 24-well plates and allowed to adhere overnight. Cells were then fixed with 4% formaldehyde solution for 15 min at room temperature, washed three times with PBS, and permeabilized with 0.3% Triton X-100 (Sigma-Aldrich, St. Louis, MO, USA) in PBS for 15 min. After additional PBS washes, cells were incubated for 1 h at room temperature in a blocking solution containing 5% bovine serum and 0.1% Triton X-100 in PBS. Cells were incubated overnight at 4 °C with primary antibodies against NANOG (Elabscience, Houston, TX, USA; E-AB-70199), SOX2 (Elabscience, Houston, TX, USA; E-AB-18159), and OCT3/4 (Elabscience, Houston, TX, USA; E-AB-68415) diluted in blocking solution according to the manufacturers’ instructions. The following day, cells were washed three times with PBS (5 min each) and incubated for 1 h at room temperature in the dark with appropriate fluorophore-conjugated secondary antibodies. After three additional PBS washes, coverslips were mounted using a DAPI-containing mounting medium (Abcam, Cambridge, UK) for nuclear counterstaining. Fluorescence images were acquired using a Zeiss fluorescence microscope (Carl Zeiss, Oberkochen, Germany) and analyzed using ZEN imaging software. For quantitative analysis, at least 500 cells were counted across multiple randomly selected microscopic fields, and the percentage of positive cells was calculated.

### Co-Staining for (Senescence Associated) SA-β-galactosidase and Ki67

Cellular senescence was assessed using the SA-β-galactosidase Staining Kit (Cell Signaling Technology, MA, USA) according to the manufacturer’s instructions. Briefly, 5,000 cells/cm² were seeded on glass coverslips in 24-well plates and allowed to adhere overnight. Cells were fixed with 2% formaldehyde for 10 min at room temperature, washed with PBS, and incubated with the β-galactosidase staining solution at 37 °C overnight.

Following SA-β-gal staining, cells were permeabilized with 0.3% Triton X-100 in PBS for 5 min on ice and subsequently incubated for 1 h at room temperature in a blocking solution containing 5% fetal bovine serum and 0.1% Triton X-100 in PBS. Cells were then incubated overnight at 4 °C with a primary antibody against the proliferation marker Ki67 (Elabscience, Houston, TX, USA; AN005400L), diluted according to the manufacturer’s instructions. The following day, after three PBS washes, cells were incubated for 1 h at room temperature in the dark with an appropriate fluorophore-conjugated secondary antibody. Coverslips were mounted using a DAPI-containing mounting medium (Abcam, Cambridge, UK) for nuclear counterstaining, and images were acquired using a Zeiss fluorescence microscope. For quantitative analysis, at least 500 cells from randomly selected microscopic fields were counted. Results were expressed as the percentage of positive nuclei.

This combined staining approach allowed discrimination among proliferating (Ki67⁺/β-gal⁻), senescent (Ki67⁻/β-gal⁺), quiescent (Ki67⁻/β-gal⁻), and stressed (Ki67⁺/β-gal⁺) cell subpopulations.

### Apoptosis Assay (Annexin V/PI)

Apoptosis was analyzed by flow cytometry using the Annexin V-FITC/Propidium Iodide Apoptosis Detection Kit (BioLegend, SAN, CA). Cells were harvested, washed, and stained as instructed by the manufacturer. The assay distinguishes viable (Annexin V⁻/PI⁻), early apoptotic (Annexin V⁺/PI⁻), and late apoptotic (Annexin V⁺/PI⁺) or necrotic (Annexin V^−^/PI⁺) populations. Early and late apoptotic fractions were quantified together under the experimental conditions.

### Reactive Oxygen Species (ROS) Detection

Intracellular ROS generation was measured using 2’,7’-dichlorodihydrofluorescein diacetate (DCF-DA; Sigma-Aldrich, St. Louis, USA). Cells were incubated with 10 µM DCF-DA, and fluorescence (excitation 488 nm; emission 525 nm) was continuously monitored for 48 h at 37 °C in a CO₂-independent medium using a GloMax microplate reader (Promega, Italia).

### Cell Viability (CCK-8 Assay)

Cell viability and proliferation were assessed using the Cell Counting Kit-8 (CCK-8; Dojindo, Munich, Germany). Cells were seeded in 96-well plates, treated as indicated, and then incubated with 10 µL of CCK-8 reagent for 2 h. Absorbance at 450 nm was measured using a microplate reader (Promega, Italia).

### Adipogenic Differentiation and Oil Red O Staining

MSCs were seeded at a density of 5,000 cells/cm² in six-well plates and cultured in complete growth medium consisting of D-MEM supplemented with 10% embryonic stem cell–qualified fetal bovine serum, 5 ng/mL recombinant human basic fibroblast growth factor, 1% penicillin/streptomycin, and 1% L-glutamine. Upon reaching 70–80% confluence, the culture medium was replaced with adipogenic induction medium composed of high-glucose DMEM containing 10% FBS, 1% penicillin/streptomycin, 1 mM dexamethasone, 10 µg/mL insulin, 0.5 mM IBMX, and 200 µM indomethacin (all from Sigma-Aldrich, St. Louis, USA). After 21 days, adipogenic differentiation was verified through Oil Red O staining for lipid droplet accumulation. Cells were washed in PBS, fixed with 10% formaldehyde for 10 min, rinsed with 3% isopropanol, stained, and then visualized by light microscopy.

### Chondrogenic Differentiation and Safranin O Staining

Chondrogenic induction was performed following a modified version of Iacono [23]. Cells (5,000 cells/cm²) were cultured in DMEM containing 1% FBS, 100 IU/mL penicillin, 100 µg/mL streptomycin, 50 nM ascorbate-2-phosphate, 0.1 mM dexamethasone, and 10 ng/mL hTGF-β1 (PeproTech, UK). The medium was renewed every 3 days. After 21 days, glycosaminoglycan synthesis was detected by Safranin O staining. Cells were fixed in cold acetone: methanol (1:1) for 3 min, stained for 30 min, rinsed, and air-dried before imaging.

### Osteogenic Differentiation and Alizarin Red S Staining

Osteogenesis was induced by culturing MSCs (5,000 cells/cm²) in high-glucose DMEM supplemented with 10% FBS, 100 nM dexamethasone, 10 mM β-glycerophosphate, and 50 µM ascorbate-2-phosphate (Sigma-Aldrich, St. Louis, USA). After 21 days, calcium deposition was detected by Alizarin Red S staining (2%, pH 4.2). Cells were washed, fixed with 4% formaldehyde for 15 min, stained for 20 min, and visualized under a bright-field microscope.

### Indoleamine 2,3-Dioxygenase (IDO) Expression

IDO activity was quantified using the IDO1 Activity Assay Kit (Abcam, Cambridge, UK; ab235936). MSCs were seeded at a density of 5,000 cells/cm² and cultured under standard growth conditions. To induce immunomodulatory licensing, cells were treated with 50 ng/mL interferon-gamma (IFN-γ) for 48 h. Following stimulation, fluorescence was measured at Ex/Em = 402/488 nm according to the manufacturer’s instructions. Data were expressed as mean fluorescence intensity (MFI) and normalized to unstimulated control samples.

### RNA Isolation and RT-qPCR

Total RNA was isolated from MSC cultures using EUROGOLD TriFast (Euroclone, Italy) according to the manufacturer’s instructions. RNA was extracted from approximately 2 × 10^5^ cells per sample. RNA concentration and purity were assessed spectrophotometrically using a NanoDrop instrument (Thermo Fisher Scientific). For reverse transcription, 1 µg of total RNA was used for cDNA synthesis employing the 5X All-In-One RT MasterMix (ABM, Richmond, BC, Canada), following the manufacturer’s protocol. Quantitative real-time PCR was performed using gene-specific primers for NANOG, OCT3/4, and SOX2 designed with Primer Express^®^ software (Applied Biosystems/Thermo Fisher Scientific, CA, USA). Real-time PCR reactions were carried out in triplicate using a BrightGreen 2X qPCR MasterMix (ABM) on a real-time PCR system. Gene expression levels were normalized to the housekeeping gene GAPDH, and relative quantification was calculated using the 2^−ΔΔCt^ method. Data are expressed as fold change relative to control samples.

### Statistical Analysis

Statistical analyses were performed using GraphPad Prism (GraphPad Software, CA, USA). Data distribution was assessed using the Shapiro–Wilk normality test. For normally distributed datasets involving multiple groups, one-way ANOVA followed by Bonferroni multiple-comparisons test was applied. Comparisons between two groups were performed using unpaired two-tailed Student’s t-test. For repeated-measure datasets, mixed-effects models (REML) were used where appropriate. Data are presented as mean ± SD, and p values < 0.05 were considered statistically significant. Each donor represented one biological replicate (*n* = 8). Where indicated, measurements were performed in technical triplicates per donor and averaged prior to statistical testing.

## Results

Eight lipoaspirate samples were collected using the Lipogems^®^ medical device from adult donors undergoing orthopedic or aesthetic procedures. Subcutaneous adipose tissue was harvested by standard liposuction techniques, as described in the Materials and Methods section. The main demographic and clinical characteristics of the donors are summarized in Table 1, including age, sex, body mass index (BMI), treatment indication, and treatment modality. All participants were in good general health, defined by the absence of inflammatory, autoimmune, or neoplastic diseases and immunosuppressive treatments, and underwent Lipogems-based regenerative or ultrasound-guided autologous transplantation procedures for orthopedic or aesthetic indications. Clinical outcome following Lipogems-based treatment was evaluated by the operating surgeon using a semi-quantitative 1–10 scale, where 1 indicates no perceived improvement and 10 indicates maximal perceived benefit. Overall, treatment effectiveness scores ranged from 7 to 10 across samples.

**Table 1:** Clinical and demographic characteristics of donors and treatment outcomes

Sample	F01	F62	F84	M50	M59	M64	M66	M89
Gender	Female	Female	Female	Male	Male	Male	Male	Male
Age (years)	23	62	40	74	65	60	58	35
BMI (kg/m^2^)	22.3	19	17.3	26	32.7	23	28.7	24
Reason for treatment	Facial aging and breast asymmetry	Lumbar and lumbosacral osteoarthritis	Muscle injury of the right shoulder, tendinosis of the left shoulder	Osteoarthritis of the hand	Bilateral coxarthrosis and L5 osteoarthritis.	Bilateral gonarthrosis and right-sided epicondylitis	Osteoarthritis of the shoulders and cervical spine	Degeneration of the first metatarsophalangeal joints of the feet.
Treatment modality	Regenerative LPG therapy	Ultrasound-guided LPG transplant	Ultrasound-guided LPG transplant	Ultrasound-guided LPG transplant	Ultrasound-guided LPG transplant	Ultrasound-guided LPG transplant	Ultrasound-guided LPG transplant	Ultrasound-guided LPG transplant
Effectiveness *	8	10	10	9	8	10	7	10

* Surgeon-assessed arbitrary 1 to 10 scale to assess treatment effectiveness

Treatment effectiveness was assessed by the operating surgeon using a semi-quantitative 1–10 scale.

MSCs were isolated and cultured as described in the Materials and Methods section. Immunophenotyping confirmed the presence of a surface antigen profile typical of ADSCs and MSCs, with detection of CD73⁺/CD90⁺/CD105⁺ cells, validating the mesenchymal identity of the stromal fraction in accordance with the ISCT (International Society for Cellular Therapy) minimal criteria for MSCs [12, 24]. Lipogems-derived samples were compared with a commercial adipose-derived MSC line used as a reference control.

**Fig. 1:**
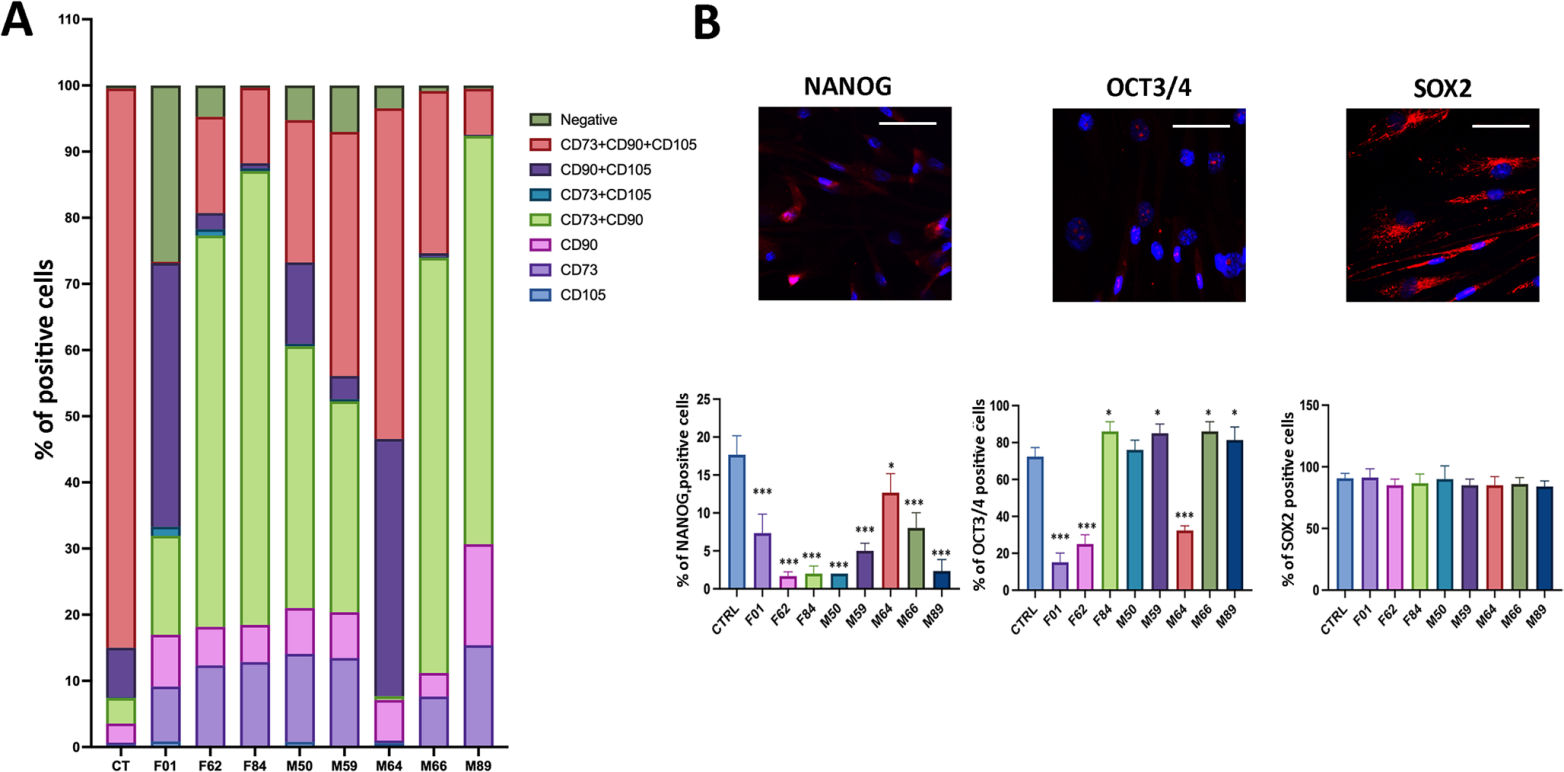
Immunophenotypic and pluripotency marker characterization of ASCs derived from lipogems lipoaspirates. (**A**) Histogram showing the percentage of cells positive for CD73⁺, CD90⁺, and CD105⁺. The graph also highlights double-positive, triple-positive, and negative populations. The proportion of triple-positive (CD73⁺/CD90⁺/CD105⁺) cells varied across donors, reflecting donor-dependent heterogeneity (age, BMI, general health) and/or differences in lipoaspiration technique rather than intrinsic senescence or apoptosis. The CD73⁺/CD90⁺ double-positive population represents pericytes and transitional pericytes, which exhibit intermediate phenotypes between pericytes and MSCs. A commercially available adipose-derived MSC line was used as reference control for marker expression. (**B**) Representative immunocytochemistry (ICC) images showing NANOG, OCT3/4, and SOX2 expression in cultured ASCs, with corresponding histograms below each image indicating the percentage of positive cells. These markers confirm the retention of pluripotency-associated transcription factors. Images were acquired at 40× magnification; the white bar in each panel represents 100 μm. (*n* = 8 donors; measurements performed in technical triplicates; mean ± SD; *p < 0.05, **p < 0.01, ***p < 0.001)

Quantitative flow cytometry analysis revealed marked inter-donor variability in the proportion of triple-positive (CD73⁺/CD90⁺/CD105⁺) cells (orange bar in Fig. 1A). In this context, samples displaying triple-positive proportions below < 20% were classified as low, whereas proportions above > 50% were considered high. Based on these criteria, sample F01 exhibited a particularly low percentage of triple-positive cells, while sample M64 showed the highest proportion within the cohort.

This variability likely reflects donor-dependent heterogeneity (e.g., age, BMI, general health) and/or differences in the lipoaspiration technique.

The double-positive CD73⁺/CD90⁺ population corresponds to pericytes and transitional pericytes, exhibiting intermediate features between pericytes and MSCs, and contributing to vascular support and tissue remodeling [25].

Consistent with previous reports on Lipogems^®^, flow cytometry of non-expanded, collagenase-treated products revealed a predominance of mature pericytes and MSCs, with only a few hematopoietic cells [26]. Our findings are in agreement with these observations, demonstrating a predominance of pericytes and MSCs (purple bar in Fig. 1A), although some exceptions were noted.

In sample M64, a reduction in pericytes was accompanied by an increase in triple-positive cells (orange bar in Fig. 1A), whereas sample F01 contained a subpopulation positive for CD105⁺ and CD73⁺, likely representing committed stem cells (turquoise bar in Fig. 1A).

Analysis of pluripotency-associated gene expression further confirmed the stem cell identity of the isolated populations. The transcription factors *OCT3/4*, *NANOG*, and *SOX2* are key regulators of pluripotency, although their expression levels can vary among individual cells.

In our samples, *NANOG* was detected in all cases but at lower levels compared with commercial adipose-derived MSC (CTRL), suggesting a partial reduction in the stabilization of the pluripotent state (Fig. 1B). *OCT3/4* expression exhibited inter-sample variability: in samples F84, M59, M66, and M89, *OCT3/4* levels were higher than in the control, whereas in M50, expression was comparable to the control (Fig. 1B). These differences may reflect subtle variations in the pluripotent potential or activation state of MSCs among donors. In contrast, *SOX2* expression remained constant across all samples, showing no significant differences relative to the control, indicating that the core pluripotent identity is preserved despite variations in *NANOG* and *OCT3/4* levels. Unstained and antibody control images used to validate immunostaining specificity are provided in Supplementary Fig. 1.

**Fig. 2:**
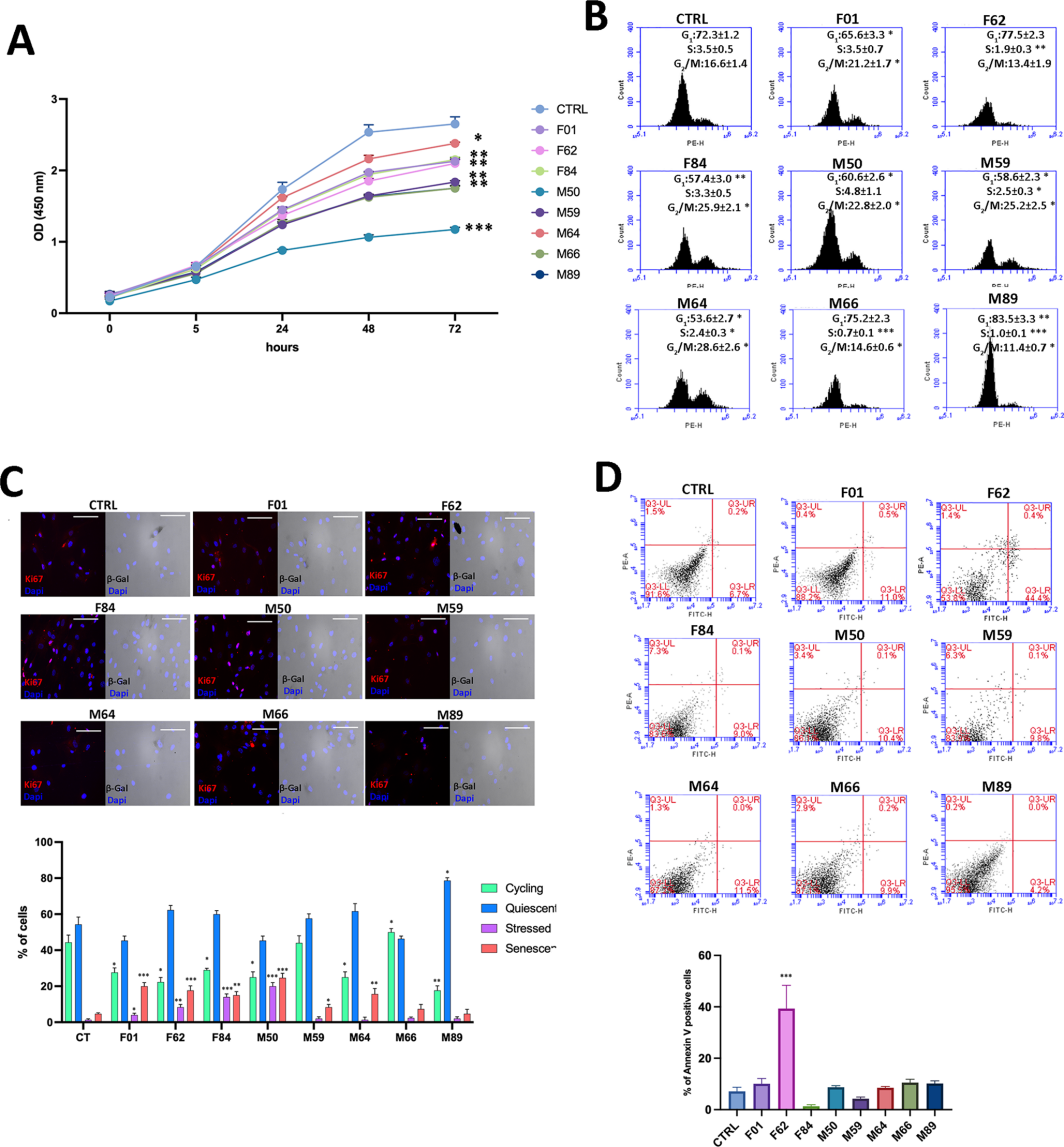
Functional assessment of proliferation, cell cycle, senescence, and apoptosis in ASC samples. (**A**) Cell proliferation evaluated by CCK-8 assay. Growth curves show optical density (OD) at 450 nm, revealing inter-sample heterogeneity in proliferative capacity. (**B**) Representative flow cytometry plots of cell-cycle distribution. Despite differences in proliferation rates, no major variation in S-phase proportions was observed, suggesting comparable cell-cycle dynamics among samples. (**C**) Representative β-galactosidase and Ki67 co-staining images. Four subpopulations were identified: double-negative (quiescent), Ki67-positive only (proliferating), double-positive (stressed), and β-galactosidase-positive only (senescent) cells. Quantitative analysis revealed no significant differences in senescence levels between samples. (**D**) Representative flow cytometry plots for apoptosis analysis. Annexin V–FITC and propidium iodide (PE channel) staining were used to identify apoptotic cells. Early and late apoptotic cells were quantified together. All samples exhibited comparable apoptotic rates, indicating that proliferative heterogeneity is independent of apoptosis or senescence. (*n* = 8 donors; measurements performed in technical triplicates; mean ± SD; *p < 0.05, **p < 0.01, ***p < 0.001)

Overall, these findings confirm the presence of a heterogeneous yet pluripotent mesenchymal population within the Lipogems-derived stromal fraction. Proliferation assays revealed marked inter-sample heterogeneity (Fig. 2A). At 72 h, optical density (OD450) values ranged from approximately 1.1 to 2.7. Sample M64 displayed the highest proliferative capacity (OD450 ≈ 2.6–2.7 at 72 h), whereas sample M50 exhibited the lowest proliferation rate (OD450 ≈ 1.1–1.2), corresponding to an approximately twofold difference in cell growth.

Cell-cycle analysis showed comparable distributions across samples, with S-phase fractions generally ranging between ~ 1–3% and G2/M fractions between ~ 14–29%, suggesting that the observed differences in proliferation may instead be due to variations in cell-cycle duration (Fig. 2B).

Senescence analysis based on combined SA-β-galactosidase and Ki67 staining revealed relatively similar proportions of senescent cells across samples (Fig. 2C). Senescent (Ki67⁻/β-gal⁺) cells generally accounted for ~ 5–15% of the population, with no sample exceeding ~ 20%. In contrast, cycling (Ki67⁺/β-gal⁻) cells showed greater variability, ranging from approximately 20–55%, with sample M66 among the highest cycling fractions and sample M89 among the lowest. These data indicate that proliferative heterogeneity primarily reflects differences in the proportion of actively cycling cells rather than accumulation of senescent cells. Apoptosis analysis by Annexin V staining revealed generally low and comparable levels across most samples, with Annexin V–positive cells typically ranging between ~ 5–15% (Fig. 2D). Notably, sample F62 represented an exception, displaying a markedly higher apoptotic fraction (approximately 35–40%) compared with all other samples and the control group. Overall, all samples exhibited similar levels of senescence and apoptosis, with no significant deviations observed. However, analysis of cycling cells revealed inter-sample heterogeneity, likely linked to differences in cellular composition. 

**Fig. 3:**
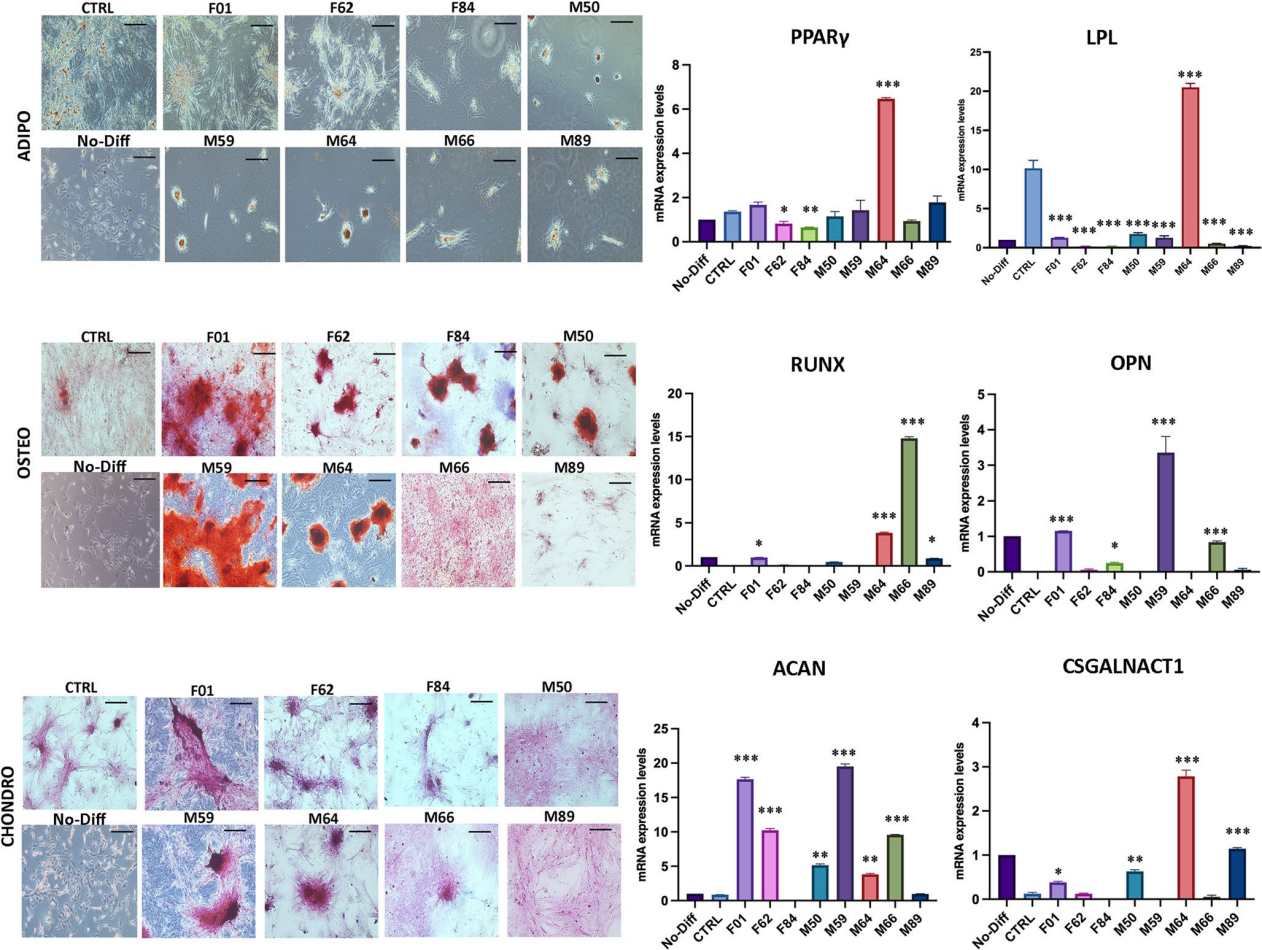
Spontaneous differentiation potential of ASCs derived from lipogems lipoaspirates. Representative images of adipogenic, osteogenic, and chondrogenic differentiation of MSCs following induction. Adipogenic differentiation was assessed by Oil Red O staining, showing intracellular lipid accumulation, with corresponding histograms indicating gene expression levels of LPL and PPARγ. Osteogenic differentiation was evaluated using Alizarin Red S staining for calcium deposition, accompanied by expression analysis of OPN and RUNX2. Chondrogenic differentiation was confirmed by Safranin O staining of glycosaminoglycans, with corresponding mRNA expression of ACAN and CSGALNACT1. All images were acquired at 40× magnification; the dark bar in each panel represents the scale bar (100 μm). (*n* = 8 donors; measurements performed in technical triplicates; mean ± SD; *p < 0.05, **p < 0.01, ***p < 0.001)

Differentiation assays indicated a general bias toward chondrogenic commitment in most samples, in contrast to commercial adipose-derived MSC line used as a reference control, which predominantly favored adipogenic differentiation. Only sample M64 exhibited pronounced adipogenic differentiation, as indicated by elevated LPL expression (Fig. 3). For transcriptional analyses, lineage-specific genes were selected to capture both early commitment and matrix-related aspects of differentiation. In the adipogenic lineage, LPL and PPARγ were used as established markers of lipid metabolism and adipocyte commitment. Osteogenic differentiation was assessed using OPN and RUNX2, reflecting extracellular matrix deposition and osteogenic transcriptional programming, respectively. For chondrogenic differentiation, ACAN and CSGALNACT1 were chosen as markers of cartilage extracellular matrix synthesis and glycosaminoglycan biosynthesis, which are particularly indicative of functional chondrogenic commitment.

**Fig. 4:**
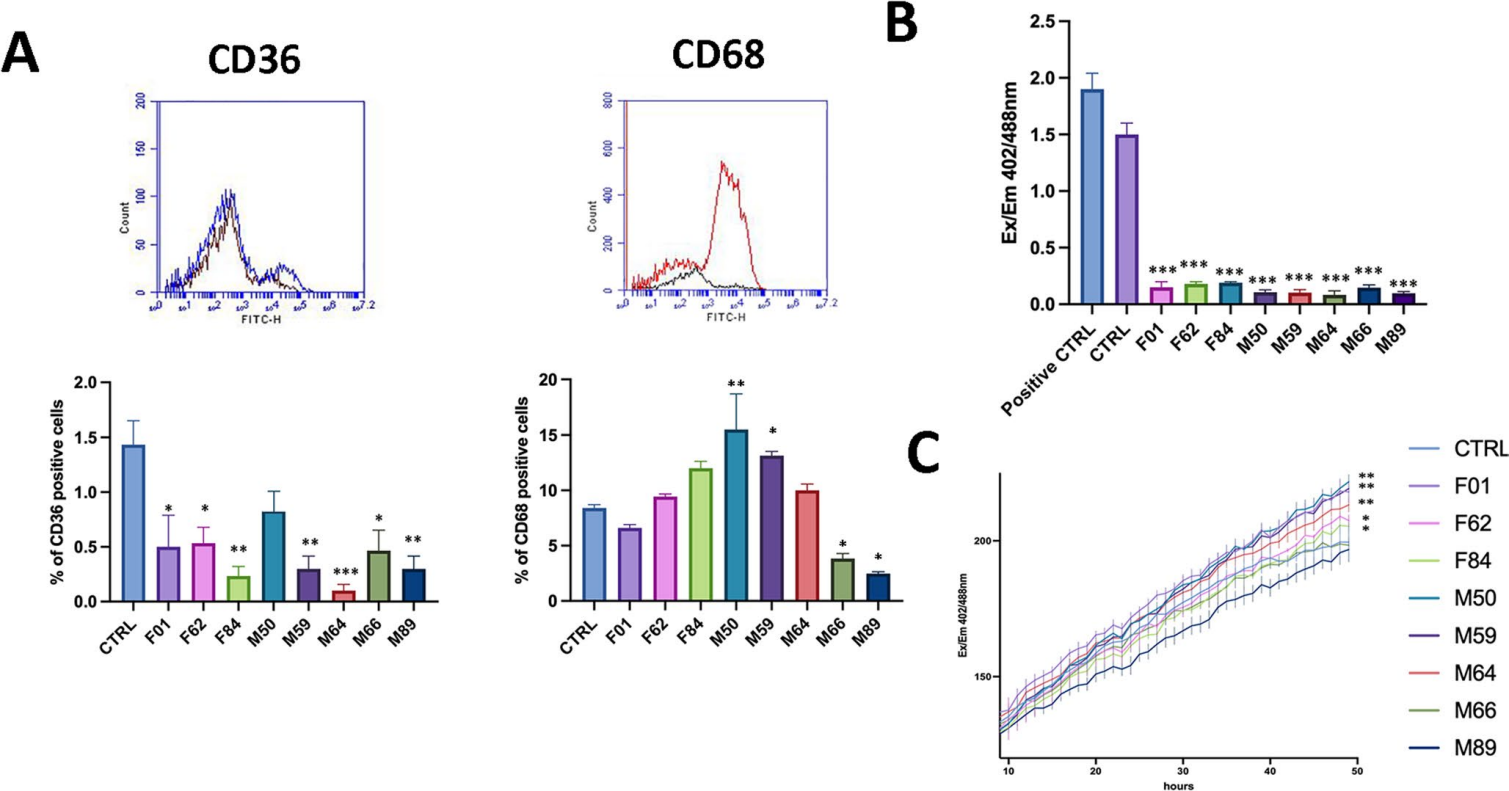
Inflammatory markers, immunomodulatory enzyme expression and reactive oxygen species (ROS) levels in ASC samples. (**A**) Representative flow cytometry plots for CD36⁺ and CD68⁺ (top) with corresponding quantification histograms shown bottom each plot. In the histograms, the black line represents unstained cells, blue and red lines represent specific antibody staining profiles. (**B**) Histogram showing IDO (indoleamine-2,3-dioxygenase) expression across samples, demonstrating generally low IDO levels under the tested conditions. (**C**) Line-plot (curve) of DCFDA fluorescence (ROS indicator) across samples. ROS levels increased in all samples except M89 and M66, which did not show an increase and correspond to the youngest donors in the cohort, suggesting a potential donor-age effect on oxidative response.(*n* = 8 donors; measurements performed in technical triplicates; mean ± SD; *p < 0.05, **p < 0.01, ***p < 0.001)

These results suggest intrinsic differences in lineage bias among samples, likely driven by variations in cellular composition rather than differences in proliferation or apoptosis. Inflammatory profiling of CD68 and CD36 revealed additional heterogeneity (Fig. 4A).

Samples F01 and M64 showed CD68 levels comparable to the control, suggesting low inflammatory activation, whereas F01 displayed reduced CD36 expression, potentially indicating a diminished lipid-mediated anti-inflammatory capacity [27]. In contrast, samples F84, M50, and M59 exhibited markedly higher CD68 expression, consistent with increased macrophage activation or infiltration. CD36 expression, however, remained variable and did not consistently correlate with CD68 levels. IDO (indoleamine-2,3-dioxygenase) expression was low across all samples (Fig. 4B), indicating limited classical immunosuppressive activity under the tested conditions.

Intracellular reactive oxygen species (ROS) levels were assessed using the 2′,7′-dichlorodihydrofluorescein diacetate (DCFDA) assay, with oxidation to fluorescent DCF (Ex 488 nm/Em 525 nm) serving as a measure of oxidative stress. Most samples exhibited elevated ROS relative to the control (Fig. 4C), observed alongside CD68 and CD36 expression and suggesting a pro-inflammatory MSC phenotype.

Notably, samples with higher ROS also displayed low IDO expression, suggesting that elevated oxidative stress may be associated with reduced immunosuppressive potential. In contrast, sample M89 showed the lowest ROS level among all samples, including the control, consistent with a more quiescent MSC state and potentially preserved immunomodulatory function.

Overall, these data underscore donor-dependent variability in oxidative stress and its impact on MSC immunomodulatory capacity.

## Discussion

In this study, we demonstrated that Lipogems-processed lipoaspirate is characterized by pronounced inter-donor heterogeneity in mesenchymal stromal cell content, stemness-related transcriptional profiles, and functional properties, highlighting intrinsic biological variability as a critical determinant of therapeutic outcomes. Despite this heterogeneity, clinical outcomes remained consistently high across all donors, suggesting that therapeutic efficacy relies on multiple complementary mechanisms rather than on any single cellular parameter.

Autologous adipose tissue has gained widespread use in aesthetic and reconstructive procedures due to its abundance, biocompatibility, and minimally invasive collection methods. While initially valued primarily for its volumetric filling capabilities, it is now recognized as a biologically active tissue with significant regenerative potential. This regenerative capacity is largely attributed to the presence of adipose-derived stem cells (ADSCs), which exhibit both multipotent differentiation potential and paracrine activity, thereby promoting tissue repair and modulating the local microenvironment [28, 29].

In recent years, ADSCs have emerged as a promising and versatile source of MSCs for both experimental and clinical applications. However, reproducibility and translational consistency remain limited, largely due to the absence of standardized procedures for ADSC isolation, characterization, and quality assessment. An additional challenge stems from the intrinsic variability of lipoaspirate, which can be influenced by multiple biological and technical factors. This cellular heterogeneity may arise from: (i) the anatomical origin of the adipose depot (e.g., subcutaneous vs. visceral fat); (ii) donor-specific characteristics, including age, sex, body mass index, and overall health; and (iii) methodological differences in study design, harvesting devices, or tissue processing protocols [29–31]. Moreover, intrinsic properties of the cells within each sample may further contribute to variability.

In this preliminary study, we performed a comprehensive molecular and cellular characterization of Lipogems-processed lipoaspirate to identify intrinsic sources of variability and their potential impact on therapeutic outcomes. Immunophenotypic profiling revealed substantial inter-donor heterogeneity in the proportion of CD73⁺/CD90⁺/CD105⁺ MSCs and pericytes, suggesting that donor-specific biological factors rather than technical variability primarily contribute to differences in stromal composition. This finding is consistent with previous reports indicating that adipose-derived stromal cell populations are highly heterogeneous and influenced by donor-related variables and tissue microenvironmental factors.

To investigate the molecular basis of this heterogeneity, we analyzed the expression of core pluripotency-associated transcription factors. OCT3/4, NANOG, and SOX2 constitute a central regulatory network responsible for maintaining stem cell identity and repressing differentiation-associated gene programs [32–34]. In our samples, NANOG was consistently expressed, albeit at reduced levels compared with controls, consistent with a partially primed pluripotent state previously reported for adult MSCs [34–36]. This reduction may indicate a shift from a naïve pluripotent state toward a lineage-primed phenotype, which is characteristic of adult stromal populations with limited self-renewal capacity.

OCT3/4 displayed marked inter-sample variability, which may reflect differences in stemness state and lineage priming [37, 38]. Variations in OCT3/4 levels have been reported to influence lineage bias, with elevated expression associated with mesoendodermal priming and reduced expression linked to ectodermal or trophoblastic differentiation pathways. In this context, the increased OCT3/4 expression observed in specific samples may indicate enhanced activation of self-renewal pathways and could be associated with a higher proportion of MSC-like cells exhibiting regenerative potential [39]. In contrast, SOX2 expression remained stable across samples, supporting the maintenance of a basal stem-like transcriptional program despite variability in OCT3/4 and NANOG expression [40]. The relative stability of SOX2 has been previously reported in adult stromal populations and may reflect its role in sustaining core stemness functions even under partial lineage commitment. Together, these observations suggest dynamic heterogeneity in stemness-related transcriptional states within Lipogems-derived stromal fractions, potentially reflecting transitions between pluripotent substates previously described in embryonic and adult stem cell populations [41].

Functional assays revealed inter-sample variability in proliferation and differentiation potential that was independent of senescence or apoptosis, indicating that intrinsic cellular composition rather than cell-cycle arrest mechanisms likely drives functional diversity. Notably, lineage bias differed among samples, supporting the concept that donor-dependent cellular composition and stemness state influence differentiation propensity. These findings are consistent with previous reports demonstrating that MSC populations exhibit donor-dependent lineage commitment profiles.

The immunomodulatory capacity of MSCs is a critical determinant of clinical efficacy in regenerative and reconstructive applications [42]. In our study, IDO expression was uniformly low, suggesting limited activation of classical immunosuppressive pathways under the experimental conditions. IDO is known to be inducible by inflammatory cytokines such as interferon-γ and modulated by glucocorticoids [43–45]; therefore, its low expression may reflect the absence of inflammatory stimuli during tissue processing or donor-dependent differences in tissue microenvironment. In addition, intrinsic donor variability, metabolic status, and local inflammatory cues may influence basal IDO activity [46, 47]. These findings highlight the potential need for targeted preconditioning strategies to enhance MSC immunomodulatory efficacy prior to clinical application.

Concurrently, we observed heterogeneity in intracellular ROS levels and inflammatory markers (CD68, CD36). CD68 is widely used as a marker of macrophage infiltration and inflammatory activation, whereas CD36 plays a role in lipid uptake, metabolic homeostasis, and resolution of inflammation in adipose tissue [48–51]. Samples with elevated ROS and CD68 expression (F84, M50, M59) displayed a pro-inflammatory phenotype with potentially reduced immunosuppressive capacity, whereas M89 showed the lowest ROS levels, consistent with a more quiescent state. Oxidative stress is known to modulate MSC phenotype and immunoregulatory functions, suggesting that donor-dependent oxidative states may contribute to variability in therapeutic outcomes. The heterogeneous expression of CD68 and CD36 further underscores donor-dependent variability in inflammatory microenvironmental conditions.

Despite molecular and cellular heterogeneity, clinical outcomes were consistently high, indicating that therapeutic efficacy may rely on multiple mechanisms, including paracrine signaling, extracellular matrix remodeling, angiogenesis, and differentiation potential, rather than solely on classical IDO-mediated immunosuppression. This observation is consistent with the concept that adipose-derived stromal fractions exert regenerative effects through a complex network of trophic and immunomodulatory signals. The Lipogems system appears to provide a standardized microenvironment that preserves native stromal architecture and cellular interactions, potentially buffering against individual cellular variability.

## Limitations

This study has several limitations that should be acknowledged. First, the number of samples included was relatively low, which may affect the statistical power and generalizability of the findings. Additionally, the study design was observational, limiting the ability to establish causal relationships. Future studies with larger cohorts and experimental approaches are warranted to validate and expand upon these results.

## Conclusion

Lipoaspirates processed with the Lipogems system exhibit significant inter-sample variability in cellular composition, stemness marker expression, and functional properties, while maintaining consistent viability. IDO expression was consistently low across all samples, suggesting a limited contribution of classical immunosuppressive pathways and indicating that further studies are needed to clarify IDO’s role in MSC-mediated immunomodulation. CD68 and CD36 expression patterns revealed sample-specific inflammatory states, highlighting donor- and microenvironment-dependent heterogeneity.

Collectively, these findings indicate that the regenerative and immunomodulatory potential of lipoaspirate-derived MSCs cannot be assumed a priori. Comprehensive, sample-specific characterization, including analysis of cellular composition, differentiation bias, inflammatory profile, and IDO activity, is essential for accurate prediction of clinical outcomes.

While this study suggests potential advantages of the Lipogems system in providing a standardized microenvironment and selectively enriched MSC subpopulations, it does not establish clear superiority over traditional liposuction-derived MSCs.

The control samples included in this study serve as a reference for conventional isolation methods, highlighting both similarities and differences in cellular behavior.

Ultimately, these results underscore the need for cautious interpretation of MSC functionality and therapeutic potential. Rigorous, donor-specific evaluation is critical not only to ensure reproducible outcomes in regenerative, reconstructive, and aesthetic medicine, but also to optimize resource allocation, reduce healthcare costs, and enable more efficient, evidence-based treatments.

Further comparative studies are warranted to delineate the specific advantages of Lipogems versus conventional approaches.

## Supplementary Information

Supplementary file 1.

## Data Availability

Not applicable.
