# Supplementary figures and images for: Molecular and Cellular Analysis of Lipogems-Processed Lipoaspirates for Evaluating the Efficacy of Treatments in Regenerative Medicine
Source: Stem Cell Rev Rep. 2026 Apr 11;22(5):2405–18. doi: 10.1007/s12015-026-11094-9 (PMC13241425; doi:10.1007/s12015-026-11094-9)

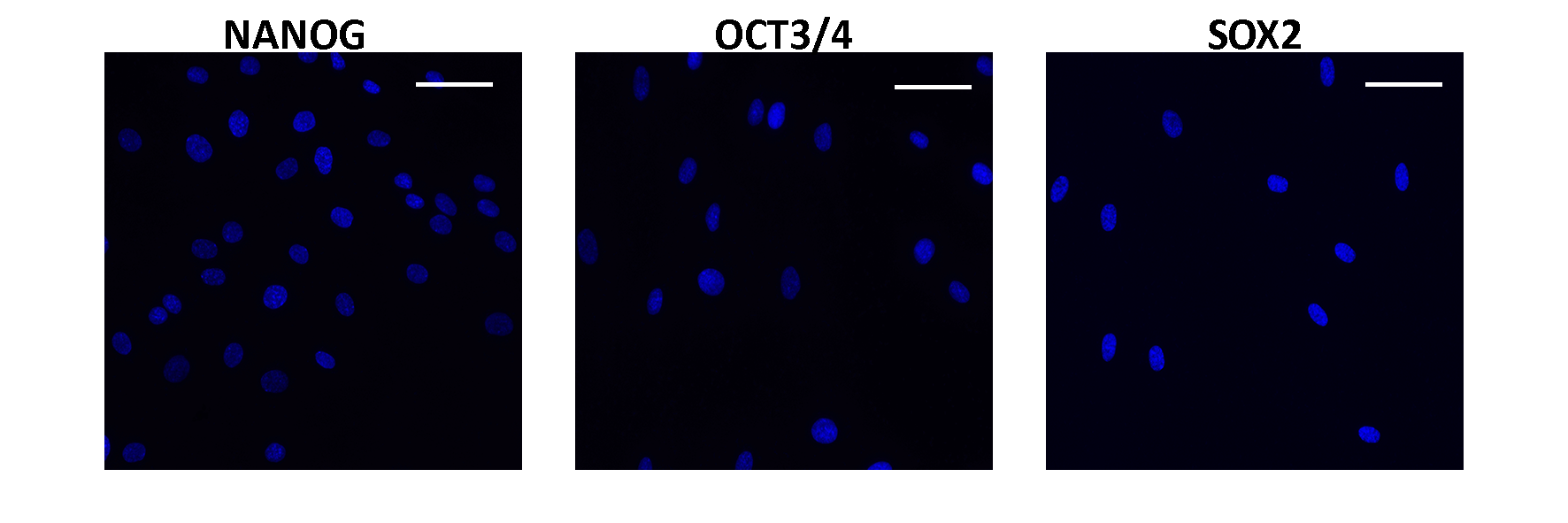

Supplement: Supplementary file 1 [file 12015_2026_11094_MOESM1_ESM.tif]
